# Supplementary material for: Conventional dendritic cells type 1 are strongly enriched, quiescent and relatively tolerogenic in local inflammatory arthritis
Source: Front Immunol. 2023 Jan 4;13:1101999. doi: 10.3389/fimmu.2022.1101999 (PMC9846246; doi:10.3389/fimmu.2022.1101999)
Supplement: Supplementary file 1 [file DataSheet_1.docx]

**Supplementary Figures:**

**
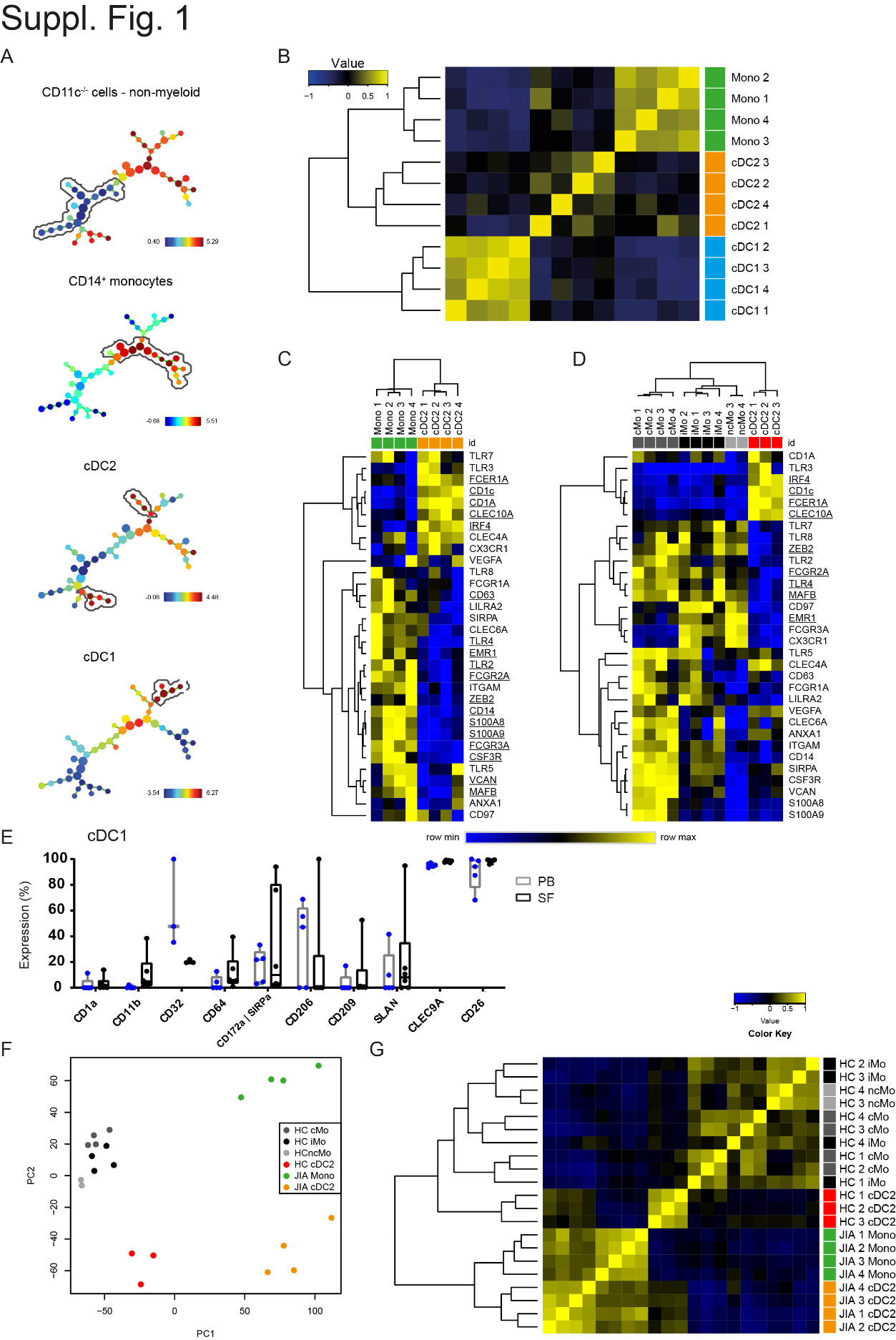
**

**Supplementary Figure 1. Related to Figure 1. Based on Flow Data.** (**A**) Unsupervised computational identification of APC populations using SPADE^1^. Expression of the marker mentioned above each subfigure is quantitatively provided by means of color, while each subset mentioned is circled in its corresponding subfigure. (**B**) Unsupervised clustering heatmap of SF APC subsets. (**C**-**D**) Heatmaps depicting relative gene expression of genes that were, based on literature, associated with monocyte-derived cells and cDC (CD141^+^ cDC-aligned markers from Fig. 1D removed) – cluster analysis performed on both rows and columns (one minus Pearson correlation, average linkage); (**C**) Comparison of SF monocytes and CD1c^+^ cDC; (**D**) Comparison of PB monocytes and CD1c^+^ cDC. cMo, classical monocyte; iMo, intermediate monocyte; ncMo, non-classical monocyte. (**E**) Comparison of monocyte-related markers and CD141^+^ cDC-related markers between paired PB and SF CD141^+^ cDC, as measured by flow cytometry (n=3 for CD32, n=5 for other markers). (**F**-**G**) PCA (**F**) and hierarchical clustering heatmap (**G**) based on variable gene expression between SF and PB monocytes and CD1c^+^ cDC in RNAseq data.

**Supplementary Figure 2. Related to Figure 2.** (**A**) Spontaneous (M) or toll-like receptor (TLR) ligand-induced cytokine production by SF APC during overnight culture. Abbreviations denote: M – medium, P3 – Pam3CSK4, PIC – Poly I:C, L – LPS, R – R848 and C – CpG.

**Supplementary Figure 3. Related to Figure 3.** (**A**) Heatmap depicting relative gene expression of co-stimulatory/-inhibitory genes that were >1 log2 RPKM and DE between SF APC – cluster analysis performed on both rows and columns (one minus Pearson correlation, average linkage).

**Supplementary Figure 4. Related to Figure 4.** (**A**) k-means clustering of SF APC RNAseq data (top panel), depicting a cluster that contain genes highly expressed by CD1c^+^ cDC and monocytes compared to CD141^+^ cDC, and GO Biological Processes (BP) terms associated with this cluster (lower panel), ranked by enrichment scores. (**B**) GO BP terms associated with CD141^+^ cDC cluster 2 genes, ranked by enrichment scores. (**C**) k-means clustering of SF APC RNAseq data (top panel), depicting a cluster that contain genes highly expressed by cDC compared to monocytes, i.e a cDC cluster, and GO BP terms associated with this cluster (lower panel), ranked by enrichment scores.


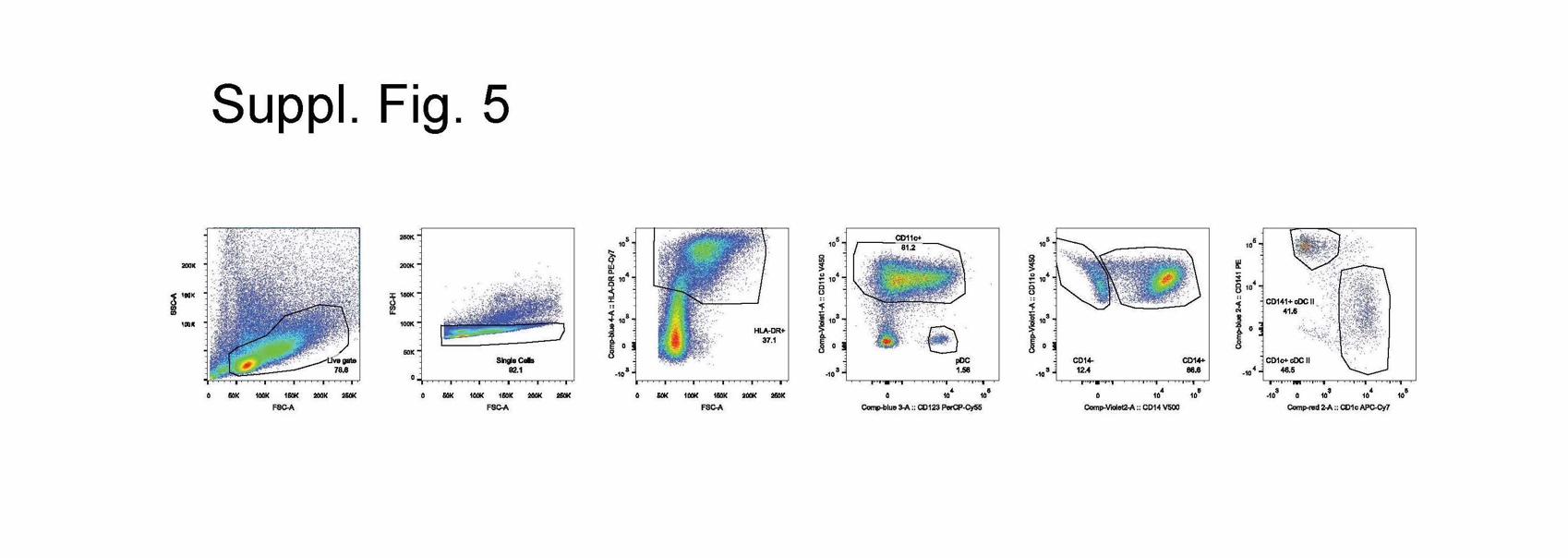


***Supplementary Figure 5. Related to Figure 1(A)*** FACS plots showing the gating strategy implemented to portray SF APC subsets. Panels from left to right indicate: all cells (FSC-A vs SSC-A), single cells (FSC-A vs FSC-H), HLA-DR^+^ cells (FSC-A vs HLA-DR PE-Cy7), CD11c^+^ cells (CD11c V450 vs CD123 PerCP-Cy55), CD14^+^ and CD14^-^ cells (CD11c V450 vs CD14 V500) and cDC1 cells (depicted in the last panel as CD141^+^ cDC II) vs cDC2 cells (depicted in the last panel as CDc1^+^ cDC II);(CD141 PE vs CD1c APC-Cy7).

**Supplementary Table 1**: Extended patient data including age, gender, type of JIA (abbreviations are: oligo-oligoarticular, poly-polyarticular, PsA- psoriatic arthritis), treatment status (abbreviations are: MTX-methotrexate, NSAID-non-steroidal anti-inflammatory drug) and (c)JADAS score – a physician-assigned, clinical score measuring overall disease activity; score included from day of sampling.

**Suppl. Table 1**

| **Patient Index** | **Age (years)** | **Gender** | **Type of JIA** | **Treatment** | **(c)JADAS Score** |
| --- | --- | --- | --- | --- | --- |
| 1 | 9 | F | Enthesistis related-JIA | MTX | 8 |
| 2 | 16 | M | Oligo | None | 7 |
| 3 | 8 | M | Oligo | MTX | 7 |
| 4 | 12 | F | Extended oligo | None | 6 |
| 5 | 19 | F | Extended oligo | Adalimumab | 6 |
| 6 | 14 | M | Extended oligo | MTX | 4 |
| 7 | 11 | F | Oligo | MTX | 4 |
| 8 | 9 | F | Oligo | MTX | 1 |
| 9 | 10 | F | Poly | None | 4 |
| 10 | 15 | M | Oligo | Unknown | N/A |
| 11 | 14 | M | Oligo | None | 3 |
| 12 | 17 | F | Oligo | None | 5 |
| 13 | 16 | M | Oligo persistent | None | 3 |
| 14 | 9 | M | Poly JIA/PsA | MTX, Adalimumab | 6 |
| 15 | 15 | M | Oligo persistent | NSAID | 9 |
| 16 | 7 | F | Oligo, Uveitis | MTX | 3 |
| 17 | 8 | F | Oligo | Leflunomide | 2 |
| 18 | 14 | F | Undetermined JIA | NSAID | 6 |
| 19 | 12 | M | Oligo | None | 4 |
| 20 | 3 | F | Oligo | None | 7 |
| 21 | 7 | F | Oligo | None | 3 |
| 22 | 8 | M | Oligo | None | 5 |
| 23 | 5 | M | Oligo | None | 4 |
| 24 | 15 | F | Oligo | None | 4 |
| 25 | 9 | F | Oligo | None | 4 |
| 26 | 11 | M | JIA, not specified further | MTX | 3 |
| 27 | 8 | M | Oligo | None | 3 |
| 28 | 11 | F | Oligo | None | 8 |
| 29 | 17 | M | Oligo | MTX | 4 |
| 30 | 4 | F | Oligo | None | 4 |
| 31 | 12 | M | Oligo | None | 3 |
| 32 | 17 | F | Oligo | None | 5 |

**Supplementary Table 2**: 1807 DE genes between SF CD1c^+^ cDC and CD141^+^ cDC, of which 780 were higher in CD141^+^ cDC, while 1027 were higher in CD1c+ cDC. There were 1260 DE genes between CD1c+ cDC and iMo, 545 and 715 of which were higher expressed in CD1c+ cDC or iMo respectively. CD141+ cDC and iMo had a higher expression of 1283 and 1577 genes, respectively, resulting in 2860 DE genes.

**Suppl. Table 2**

|  | CD1c vs. CD141 | CD1c vs. Mono | CD141 vs. Mono |
| --- | --- | --- | --- |
| CD1c high | 1027 | 545 | - |
| CD141 high | 780 | - | 1283 |
| Mono high | - | 715 | 1577 |
| *Total DE* | *1807* | *1260* | *2860* |

**Supplementary Information – Methods & Materials**

*PBMC and SFMC isolation*

Peripheral blood (PB) was drawn via veni puncture. Synovial fluid (SF) was collected during therapeutic joint aspiration. Paired PB and SF samples were taken from the same patient at the same time. SF was first treated with hyaluronidase for 30min (37°C, 5% CO_2_) to reduce the viscosity, followed by centrifugation (4°C, 1600rpm). Supernatants were removed for an additional centrifugation step (4°C, 3000rpm) to acquire SF plasma, while cell pellets were used for further SF mononuclear cells (SFMC) isolation. PB was first centrifuged (37°C, 1200rpm) to acquire PB plasma, while cell pellets were subsequently used for PB mononuclear cells (PBMC) isolation. Both SF and PB plasma were frozen at -80°C until later use. In order to obtain SFMC and PBMC, cell pellets were resuspended in RPMI containing 1% penicillin/streptomycin and cells were isolated using Ficoll-Paque density gradient centrifugation (GE Healthcare Bio-Sciences, AB) and were used either directly, or frozen in RPMI medium containing 20% FCS (Invitrogen) and 10% DMSO (Sigma-Aldrich) until further experimentation.

*Flow cytometric characterization and sort procedure of APC*

To characterize the distribution and phenotype of APC subsets in PB and SF, fresh PBMC and SFMC were stained with antibodies against HLA-DR (Clone: G46-6; BD Pharmingen or L243; Biolegend), CD11c (3.9; eBioscience or B-ly6; BD Biosciences), CD123 (7G3, BD Biosciences), CD14 (M5E2; BD Biosciences), CD1c (AD5-8E7; Miltenyi or L161; Biolegend), and CD141 (M80; Biolegend or AD5-14H12; Miltenyi) to discern the respective APC subsets: pDC (HLA-DR^+^, CD11c^-/lo^, CD123^+^; not shown), monocytes (HLA-DR+ CD11c^+^ CD14^+^), CD1c^+^ cDC (HLA-DR^+^ CD11c^+^, CD14^-^, CD1c^+^ CD141^-/lo^) , and CD141^+^ cDC (HLA^-^DR^+^ CD11c^+^, CD14^-^, CD141^+^ CD1c^-^), as shown in Figure 1A. Additional cellular hierarchy analysis from this cytometry data was performed using SPADE (Figure S1A), as described by its developers^2^. Specific settings used: FSC, SSC, CD141, CD1c, CD11c, CD14 as overlapping markers used for SPADE tree; apply compensation matrix in FCS header; Arcsinh with cofactor 150; number of desired clusters: 50.

Further phenotyping of subset markers (as shown in Figure 1E-F, 1H-I, S1G) and co-stimulatory molecules, and MHC molecules (Figure 3E) of each subset was performed using antibodies against CD1a (HI149; BD Biosciences), CD11b (ICRF44; eBioscience), CD32 (FLI8.26; BD Pharmingen), CD64 (10.1; Biolegend), CD172a (SE5A5; Biolegend), CD206 (19.2; BD Biosciences), CD209 (DCN46; BD Biosciences), SLAN (DD-1; Miltenyi), CLEC9A (8F9; Miltenyi), CD26 (2A6; eBioscience), CD40 (5C3, eBioscience), CD80 (L307.4, BD Biosciences), CD86 (IT2.2), and HLA-DR (L243, both Biolegend) and subsequently acquiring on FACSCanto II (BD Biosciences). Analysis was done using FlowJo version 10 (FlowJo, LLC).
To obtain APC subsets for functional assays or sequencing, above subset staining and gating was used to sort APC subsets on a FACSAria II or III (BD). In case of sorting APC subsets for RNAseq purposes, cells were additionally stained with antibodies against CD3 (OKT3; Biolegend), CD19 (HIB19; Biolegend), CD56 (HCD56; Biolegend) to gate out lymphocyte lineages before applying abovementioned gating strategy. After sorting, cells were washed in MACS buffer. If used directly for functional experiments, cells were resuspended in culture medium (RPMI 1640 (Lonza), 10% FCS). If intended for RNAseq purposes, sorted cells were dissolved in TRIzol (Invitrogen) and frozen at -80°C until further use.

*APC TLR stimulation and cytokine measurement*

To measure cytokine production by SF-derived monocytes and dendritic cells subsets (Figure 2F-G, and Supplementary Figure 2A), APC subsets were sorted by flow cytometry as described above and 10.000 cells were then cultured in 100µl culture volume. Cells were either not stimulated or stimulated with Pam3CSK4 (100ng/ml), Poly(I:C) (25µg/ml), LPS (100ng/ml), CpG-A (5µg/ml; all Invivogen), or R848 (1µg/ml; Enzo). After overnight culture supernatants were collected and stored at -80°C until analysis.

*Proliferation assays*

To assess the T-cell proliferation- and cytokine production-inducing capacity of APC subsets (Figure 3B-C), patient PB and SF samples were split into two fractions using CD3 magnetic-activated cell sorting (MACS) microbeads (Miltenyi Biotec), yielding CD3^+^ T cells, and CD3^-^ cells. CD3^-^ cells were subsequently stained for sorting APC subsets as described above. PB CD3^+^ T cells were labeled with 2µM cell tracer (Invitrogen) for 7 minutes at 37°C and extensively washed before used in proliferation assays. 50.000 CD3^+^ T cells were co-cultured with 10.000 SF-derived monocytes or DC subsets. At day 5, supernatants were collected to measure cytokine production, and cells were harvested, washed, and stained for T cell markers CD3 (Clone: SK7; BD), CD4 (RPA-T4; Biolegend) and CD8 (SK1; BD Biosciences). Proliferation of T cells (Figure 3B) was acquired by flow cytometry on a FACSCanto II (BD) and analyzed using FlowJo version 10 (FlowJo, LLC).

To measure APC-induced T-cell cytokine production (Figure 3C), supernatants were collected from proliferation assays and stored at -80°C until analysis.

*Luminex*

Cytokine concentrations in PB plasma, SF plasma (both in Figure 4D), APC O/N stimulation supernatants (Figure 2F-G, and Supplementary Figure 2A) and APC-T cell co-culture supernatants (Figure 3C) were measured by Luminex technology as previously described^3^.

**References (Supplementary Methods & Materials)**

1. Qiu P, Simonds EF, Bendall SC, Gibbs Jr KD, Bruggner RV, Linderman MD et al. Extracting a cellular hierarchy from high-dimensional cytometry data with SPADE. *Nat Biotechnol* 2011;29:886-91.
2. Anchang B, Hart TDP, Bendall SC, Qiu P, Bjornson Z, Linderman M et al*.* Visualization and cellular hierarchy inference of single-cell data using SPADE. Nat Protoc 2016;11:1264-79.
3. de Jager W, Hoppenreijs EPAH, Wulffraat NM, Wedderburn LR, Kuis W, & Prakken BJ*.* Blood and synovial fluid cytokine signatures in patients with juvenile idiopathic arthrithis: a cross-sectional study. Ann.Rheum.Dis 2007;66:589-598.
